# Raising community awareness to improve access to mental health services in Bali

**DOI:** 10.1192/j.eurpsy.2022.1774

**Published:** 2022-09-01

**Authors:** C.B.J. Lesmana, A. Bikker, N. Tiliopoulos

**Affiliations:** 1 Udayana University, Psychiatry, Denpasar, Indonesia; 2 The University of Edinburgh, Population Health Sciences, Edinburgh, United Kingdom; 3 The University of Sydney, School Of Psychology, NSW, Australia

**Keywords:** mental health, community awareness, access, Bali

## Abstract

**Introduction:**

Changing attitudes and behaviour regarding mental health and help-seeking is a complex process, especially in poorer areas where access to mental health services is relatively new. Data from the Indonesian national health survey indicate that after the introduction of the Universal Health Coverage a large number of people suffering from mental illness remain untreated

**Objectives:**

This study aims to address this issue by seeking the views of community leaders (i.e. village and banjars leaders) on ways to raise community awareness to improve access to mental health services, increase service utilisation rates and reduce the duration of untreated mental illness in Bali.

**Methods:**

This is a qualitative study with community leaders (i.e. village and banjar leaders) in communities in Bali on barriers and facilitators of accessing mental health services for people.

**Results:**

In Bali they still have faith in the traditional healer so if they see one and the mentally ill patient is getting better then they don’t think they need to go to the hospital. According to the community leaders the determinants for non-uptake of mental health services were mental health awareness should be integrated systematically starting at primary care and must be complemented by secondary care, and have linkages to informal community-based services and self-care. The community leaders can play a role in awareness-raising by empowerment the community and other logics in community care setting.

**Conclusions:**

Community awareness can improve access to mental health services, increase service utilisation rates and reduce the duration of untreated mental illness in Bali

**Disclosure:**

No significant relationships.

